# A hybrid recognition system for off-line handwritten characters

**DOI:** 10.1186/s40064-016-1775-7

**Published:** 2016-03-22

**Authors:** Gauri Katiyar, Shabana Mehfuz

**Affiliations:** Department of Electrical Engineering, Jamia Millia Islamia, New Delhi, India; ITS Engineering College, 46 Knowledge Park, Greater Noida, Uttar Pradesh 201308 India

**Keywords:** Character recognition, Multi layer perceptron, Feature extraction, Genetic Algorithm

## Abstract

Computer based pattern recognition is a process that involves several sub-processes, including pre-processing, feature extraction, feature selection, and classification. Feature extraction is the estimation of certain attributes of the target patterns. Selection of the right set of features is the most crucial and complex part of building a pattern recognition system. In this work we have combined multiple features extracted using seven different approaches. The novelty of this approach is to achieve better accuracy and reduced computational time for recognition of handwritten characters using Genetic Algorithm which optimizes the number of features along with a simple and adaptive Multi Layer Perceptron classifier. Experiments have been performed using standard database of CEDAR (Centre of Excellence for Document Analysis and Recognition) for English alphabet. The experimental results obtained on this database demonstrate the effectiveness of this system.

## Background

Machine simulation of functional perspective of human being has been a significant and vital research area in the field of image processing and pattern recognition (Plamondon and Srihari 2000; Arica and Yarman-Vural 2001). Handwriting Recognition is the mechanism for converting the handwritten text into a notational representation. It is a special problem in the domain of pattern recognition and machine intelligence. Character recognition can be split up into two modes: Online and offline—depending on the type of available data. Online character recognition involves the identification of character while they are being written and are captured by special hardware (e.g. smart pen or pressure sensitive tablets), which is capable of measuring pen’s pressure and velocity. Offline character recognition on the other hand, are written on paper with ordinary pens and converted into scanned digital images. Systems for recognizing machine printed text originated in the late 1950s and have been in widespread use on desktop computers since the early 1990s. In the early 1990s, image processing and pattern recognition are efficiently and effectively combined with artificial intelligence and statistical technique that is Hidden Morkov Model (HMM) (Avi-Itzhak et al. 1995; Chen et al. 1994).

Off-line handwritten character recognition continues to be an active research area towards exploring the newer techniques because it has various applications such as postal sorting, bank cheque amount reading, and official document reading. Mori et al. (1992) presented historical review of off-line character recognition research and development. A comprehensive survey of recognition techniques has been reported in (Govindan and Shivaprasad 1990; Mehfuz and Katiyar 2012; Katiyar and Mehfuz 2012). A survey on on-line and off-line handwritten and hand printed character recognition has already been presented (Plamondon and Srihari 2000). Recognition of cursive handwritten character is a difficult task. Most of the researchers have conducted their work on unconstrained character (Hanmandlu et al. 2003; Saurabh and Singh 2011; Lee 1996).

Feature selection is a process of selecting only significant features from a large database to create subset of the original database. This process retains the original feature without changing the features domain. The main objectives of the feature selection are: to reduce database dimensionally, remove irrelevant features, reduce time needed to learn the classification function, increase accuracy and to keep only important features that give comprehensive understandings for all variables. Demand of feature selection is currently rising because of the expanding sizes of databases in various applications. There are many ways of feature selection using search algorithms such as Sequential Forward Selection (SFS), Sequential Backward Selection (SBS), Exhaustive search and Genetic Algorithm (GA). Exhaustive search is not suitable for large database as it is a time consuming process. SFS and SBS operations are good because all features in database are evaluated at the same time. However once a feature is removed it does not have chance of being selected again. The selected features will remain in the selection even after new features selected, resulting some redundancies in some cases. GA differs from this type of feature selection method due to the capability to evolve new features from the selected features and a vast exploration of search space for new fitter solutions. The evolving process is made possible using GA operators which are selection, cross-over and mutations. The process will continue until the best solutions are found or the maximum number of iterations is met.

De Stefano et al. (2002) have investigated the possibility of using a pre-classification technique for reducing the number of classes. They have used genetic programming for pre classification. Parkins and Nandi (2004) have used Genetic Algorithm based feature selection and Neural Network as a classifier for handwritten digit recognition. In Saurabh and Singh (2011) performance of feed-forward Neural Networks for recognition of handwritten English alphabet has been evaluated with three different soft computing techniques namely back-propagation, evolutionary algorithm and hybrid evolutionary algorithm.

The authors have presented a GA based feature selection algorithm for detecting feature subsets, where the samples belonging to different classes are properly classified (De Stefano et al. 2013). Das et al. (2012) have applied GA to extract the optimal feature set that has the best discriminating features to recognise the handwritten Bangla digits. To recognise handwritten Farsi/Arabic digit, MLPNN (Multilayer Perceptron Neural Network) with back-propagation as classifier has been used (Shayegan and Chan 2012). By employing 1 and 2D spectrum diagram for standard deviation and minimum to maximum distribution, an optimal subset of initial feature subset was selected automatically.

The authors have proposed a fast and efficient solution based on Genetic Algorithm to solve the feature selection problems (Chouaib et al. 2012; Lanzi 1997). Lp-norms generalized principle component analysis (PCA) for dimensionality reduction has been proposed in (Liang et al. 2013).

To recognise Chinese character a Genetic Algorithm based feature selection method with Support Vector Machine as a classifier has been proposed (Feng et al. 2004). A multi objective Genetic Algorithm has been used for feature selection for handwriting recognition (Oliveira et al. 2002). The authors have proposed a new feature selection method for multi class problem based on recursive feature elimination in least square Support Vector Machine (LS-SVM) (Zeng and Chen 2009). In another work the authors have proposed a simple multilayer cluster neutral network with five independent sub networks for off-line recognition of totally unconstrained handwritten numeral and Genetic Algorithm have been used to avoid problems of finding local minima in training the multilayer cluster neural network with gradient descent techniques (Lee and Kim 1994; Lee 1995, 1996). Comparison of five feature selection methods using Neural Network as a classifier for handwritten character recognition has been presented in Chung and Yoon (1997). In Cho (1999) GA determines the optimal weight parameter of the neural network to classify the unconstrained handwritten numerals.

In our work GA has been used for feature selection because of its potential as an optimization technique for complex problems. Advantage of this approach include the ability to accommodate multiple criteria such as number of features and accuracy of classifier as well as the capacity to deal with huge database in order to adequately represent the pattern recognition problem.

The decision making part of the recognition system is the classification stage. The efficiency of the classifier depends on the quality of the features. Different classification techniques for character recognition can be split up into two general approaches (Plamondon and Srihari 2000; Arica and Yarman-Vural 2001).

### Classical techniques

Template matching, Statistical techniques and Structural techniques

### Soft computing techniques

Artificial Neural Networks (ANNs), Fuzzy logic technique, and Evolutionary computing techniques.

Nowadays Classical techniques are not extensively used in this area of research because the performance of classical techniques are dependent on the amount of data used in the defining the parameters of statistical model and are not flexible enough to adapt to new handwriting constraints. Moreover these techniques can be used only when the input data is uniform with respect to time. Recognition techniques based on Neural Network has been currently taking more attention of researchers. Quick recognition, automatic learning and flexibility once the networks are properly trained are some of the advantages of ANN. Therefore we have chosen Neural Network as a classifier in our recognition system.

## Contribution of this work

To improve the performance of the off-line handwritten character recognition system either the performance of the classifier has to be improved or better feature extraction techniques and/or feature selection techniques need to be explored. We have presented a methodology for hybrid feature extraction and a Genetic Algorithm based approach for optimal selection of feature subset along with an adaptive Multi layer perception (MLP) as pattern classifier. Adaptive nature in the classifier is achieved by implementing a function for selection of best architecture of MLP during the feature selection and classification phases. We have extracted seven feature sets based on moment features, distance-based feature, geometrical feature and local features as discussed in the following section. The effectiveness of the method is tested on the problem of off-line handwritten character recognition to address the problem of diversity in style, size and shape, which can be found in handwriting produced by different writers. All the handwritten data considered here are unconstrained alphabets to avoid the process of segmentation. Selection of features also plays an important role in improving the performance of the system. The novelty of the proposed recognition system is that by hybridization of the feature extraction techniques and randomly selecting the features using GA along with an adaptive MLP Neural Network classifier, the accuracy of the system is improved and the computational time of the system is reduced.

## Proposed recognition system

The objective of the proposed work is to use a GA based feature reduction technique to develop an off-line character recognition system using Adaptive Neural Network as a classifier. The following steps have been adopted in the algorithm for character recognition:

- Input
- Pre-processing
- Feature extraction
- Feature selection using GA
- Artificial Neural Network based classifier.

**Fig. 1:**
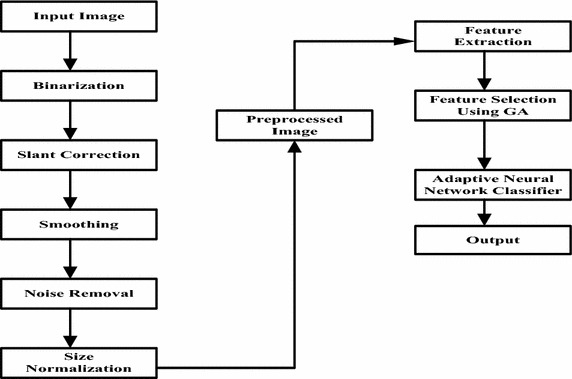
Block diagram of the proposed recognition system

All the above mentioned stages and their interconnection for the proposed recognition system are shown in Fig. 1.

### Input database

For off-line handwritten English alphabets recognition the benchmark dataset used by researchers is CEDAR (Center for Excellence in Document Analysis and Recognition, USA) CDROM-1. The database has been acquired from CEDAR, Buffalo University, USA (http://www.cedar.buffalo.edu/ilt/research.html). The bi-tonal images of alphabetic and numeric characters in the database are divided into two groups. One group contains mixed alphabetic and numeric (BINANUMS) and the other group contains numeric characters (BINDIGIS) only. The separation of database into training and testing set is shown in Table 1 (Hull 1995). There are 19,145 known alphabet characters in training set and 2183 unknown alphabet characters in testing set.

**Table 1:** Number of samples from CEDAR CDROM-1for training and testing

Capital alphabet	Training set	Testing set	Small alphabet	Training set	Testing set
A	1237	162	a	57	68
B	595	69	b	84	8
C	588	51	c	211	17
D	388	49	d	249	26
E	490	57	e	736	107
F	287	32	f	120	16
G	143	19	g	93	14
H	247	25	h	205	23
I	490	56	I	803	70
J	68	15	j	1	2
K	160	19	k	94	14
L	563	87	i	684	69
M	588	59	m	184	21
N	1022	123	n	469	60
O	905	102	o	833	84
P	516	59	p	129	7
Q	3	2	q	5	2
R	749	97	r	460	47
S	834	77	s	490	29
T	441	54	t	428	46
U	268	31	u	333	26
V	201	27	v	127	20
W	249	30	w	102	15
X	112	19	x	173	10
Y	259	39	y	136	15
Z	24	7	z	15	0
Total	11,454	1367	Total	7691	816

### Preprocessing

The main goal of the pre-processing is to arrange the information to make the character recognition process simpler. The following pre-processing steps have been applied.

#### Binarization

In order to avoid the problems resulting due to noise and lost information, the gray scale image of up to 256 gray levels is converted into binary matrix. We have used global thresholding method for binarization. If the intensity of the pixel is more than a particular threshold value, it is set to white (represented by 1) and otherwise to black (represented by 0). In this work we have chosen **mean [mean (image)]** as a threshold. The threshold value changes as the images change.

#### Slant correction

Slant is defined as slope of the general writing trend with respect to the vertical line. The image matrix is divided into upper and lower halves. The centers of gravity of the lower and upper halves are computed and connected. The slope of the connecting line defines the slope of the window (image matrix) (Hanmandlu et al. 2003; Hanmandlu and Murthy 2007).

#### Smoothing and noise removal

Exact surrounding region of a character image can be determined by smoothing using Wiener filter. Median filter is applied to further enhance the image quality by removing any leftover noise.

#### Normalization

**Fig. 2:**
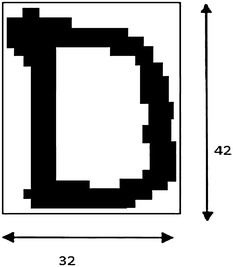
Normalisation of an image into a window size of 42 × 32

Normalization is required as the size of the character varies from one person to another and also from time to time even when the person is same. Normalization helps in equating the size of the character image (binary matrix) so that features can be extracted on the same footing. The character image is standardized to a window size of 42 × 32 as shown in Fig. 2.

### Feature extraction and feature selection

For any recognition system, feature extraction is an integral part. There are several feature extraction techniques and its selection is the most crucial factor in achieving high accuracy. The right choice of the features from the whole available set is the most important step in any classification process. Different feature selection approaches have been proposed in literature (Trier et al. 1996).

#### Feature extraction

At this stage, the features of the characters that are crucial for classifying them at recognition stage are extracted. We have extracted seven sets of feature vectors. The methods of feature extraction are as follows:

##### Box approach

**Fig. 3:**
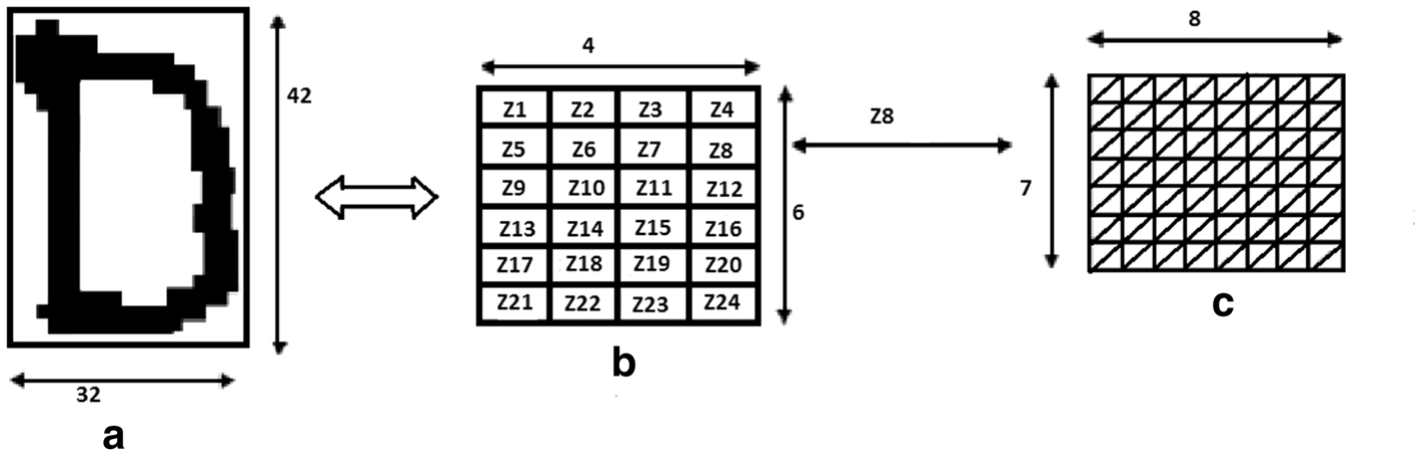
Diagonal distance feature extraction method

Box approach is based on spatial division of character image. Horizontal and vertical grid lines of 6 × 4 are superimposed on the character image of size 42 × 32. In this process 24 boxes, each of size 7 × 8 are devised. Some of the boxes will have a portion of the image and others remain empty. However, all boxes are considered for analysis (Hanmandlu et al. 2003; Hanmandlu and Murthy 2007) as shown in Fig. 3.

A normalized vector distance for each box is computed as:

1 $$\gamma_{b} = \frac{1}{{n_{b} }}\sum\limits_{k = 1}^{{n_{b} }} {d_{k}^{b} }$$

2 $$d_{k}^{b} = (i^{2} + j^{2} )^{{\frac{1}{2}}}$$

where n_b_ is number of pixels in bth box.

Normalized angle for each box is computed as:

3 $$\alpha_{b} = \frac{1}{{n_{b} }}\sum\limits_{k = 1}^{{n_{b} }} {\theta_{k}^{b} }$$

where b = 1, 2,…,24. θ_k_ = tan^−1^(−j/i) for pixel at (i, j).

These 24 pairs constitute the feature set.

##### Diagonal distance approach

Every character image of size 42 × 32 pixels is divided into 24 equal zones each of size 7 × 8 pixels. The features are extracted from each zone pixels by moving along the diagonals of its respective 7 × 8 pixels. Each zone has 14 diagonal lines, thus 14 sub-features are obtained from the each zone. These 14 sub-features values are averaged to form a single feature value and placed in the corresponding zone. Finally, 24 features are extracted for each character (Pradeep et al. 2011). The complete process is shown in Fig. 3.

##### Mean

Mean gives an idea of what pixels color to choose to assess the color of the complete image. It is measured by taking sum of all the 1 pixels and dividing them by total number of pixels of each box. Thus 24 features of each image are obtained.

The mean for each box is commutated as:

4 $$Mean = \bar{X} = \frac{{\sum\limits_{i = 1}^{n} {X_{i} } }}{N}$$

where N = Total number of pixels in each box.

##### Gradient operations

Image gradient is the variations of pixels in horizontal and vertical directions. Image gradient may be used to extract information from images. The gradients have been calculated by using the following formula.

5 $$Gradient = \nabla F = \frac{\nabla f}{\nabla x}i + \frac{\nabla f}{\nabla y}j$$

∆*f*/∆*x* = Gradient in the x-direction for pixel at (i, j). ∆*f*/∆*y* = Gradient in the y-direction for pixel at (i, j).

Gradient have been measured for each box. This process is sequentially repeated for all the 24 boxes. Thus 48 features are extracted from 24 boxes.

##### Standard deviation

Standard deviation (SD) of pixels in each box has been calculated. This process is applied for all 24 boxes to obtain 24 features.

6 $$SD = \sigma = \sqrt {\frac{1}{N}\sum\nolimits_{i}^{N} {(x_{i} - \bar{x})^{2} } }$$

where N is number of rows and $\overline{x}$ is mean.

##### Center of gravity (CG)

Centre of gravity of the pixels in each box is obtained. In this process we get total 48 features for all the 24 boxes

7 $$CG:x_{o} = \frac{{\sum\nolimits_{i = 1}^{n} {\sum\nolimits_{j = 1}^{m} {iB(i,j)} } }}{a},y_{o} = \frac{{\sum\nolimits_{i = 1}^{n} {\sum\nolimits_{j = 1}^{m} {jB(i,j)} } }}{a}$$

where *B*(*i*, *j*) is Binary Region and is given as: $B\left( {i,j} \right) = 1 if \left( {i,j} \right)\,in \,region\, or\, 0 \,otherwise.$ a = Area.

##### Edge detection

Edge detection is performed on each box and finds the sum of values at edge pixel positions. Sobel edge detection function has been used for this step. The magnitude of the gradient of the image is calculated for each pixel position in the image according to the following formula

8 $$Mag(\nabla F) = \sqrt {(F_{x} )^{2} + (F_{y} )^{2} }$$

where *F*_*x*_ = Gradient in x direction; *F*_*y*_ = Gradient in y direction. This process is applied for all 24 boxes to get 24 features.

Thus with the help of all the seven feature extraction methods we obtain 240 number of features for each image matrix.

##### Hybrid features

We have obtained a set of 240 features from seven different approaches. Instead of using a single approach we have combined all features obtained by different methods. This combination forms hybrid feature set and is stored in a feature vector. This vector contains a total of 240 features set and is used for recognizing the character. Table 2 specifies the number of features extracted by using various feature extraction techniques.

**Table 2:** Features description

Feature set	Number of features
Box features	48
Diagonal distance	24
Mean	24
Gradient operations	48
Standard deviation	24
Centre of gravity	48
Edge detection	24
Total	240

#### Feature selection

**Fig. 4:**
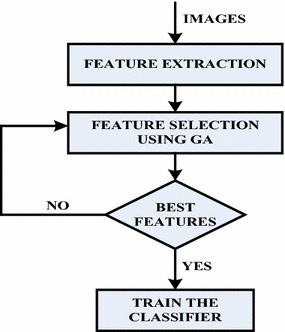
Flow chart for feature extraction and feature selection sub system

In this work GA has been used for feature selection. Figure 4 shows the basic steps of feature selection subsystem.GA is implemented on hybrid features to create a subset of best features. Feature selection can be classified into two main approaches: filter and wrapper approaches. In filter approach evaluation is normally done independently of any classifier. We have used wrapper approach for implementing GA to evaluate feature subset. Wrapper approach employs classifier’s predictive accuracy for the evaluation of the subset of features. The classifier used here is ANN, which imparts a hybrid nature to the feature selection process.

##### Procedure to prune the feature set dimensionally

**Fig. 5:**
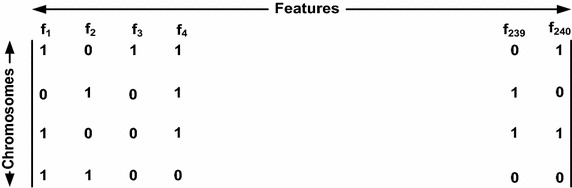
Initial populations

**Fig. 6:**
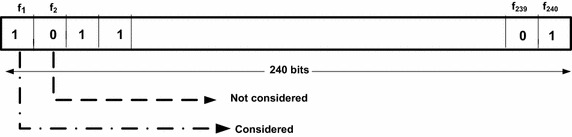
Bit string encoding

For initial population we have chosen four chromosomes where size of each chromosome is equal to the dimensionality of the feature set, which is equal to 240 as we have extracted 240 features set in the feature extraction step. In a chromosome, 1 represents presence of that particular dimension and 0 represents absence of the same (see Fig. 5). Bit string encoding of each chromosome has been shown in Fig. 6.

For each chromosome we have formed the relevant feature set say F1, F2, F3 and F4 (see Table 3).

**Table 3:** Relevant features of chromosome

Chromosome	Relevant feature
1	F1
2	F2
3	F3
4	F4

By using these relevant feature set, we performed training of Neural Network and calculated the accuracy which has been considered as the fitness function of that particular chromosome. Then we have calculated the average of fitness values of all the chromosomes. This process continues until convergence. To get the new sets of chromosomes the fittest chromosomes undergo reproduction, cross over and mutation process. Again for each new chromosome we form relevant feature set and train the Neural Network and calculate the accuracy using Eq. (9), which is the fitness function. The average of all the fitness function has been calculated. After convergence the relevant feature set from the chromosome (fittest one) has been chosen to prune the feature dimension. The various parameters of GA have been shown in Table 4.

**Table 4:** Parameters of GA

GA parameters	Value
Elitism size	1
Population size	240
Initial population	4
Selection method	Roulette-wheel
Crossover probability	0.8
Crossover point	Random
Mutation probability	0.01
Generation number	Till convergence

9 $$\% Accuracy = Fitness \,function = \frac{Number\,of\,correctly \,recognised \,character}{Total\, number\, of\, character }$$

Following are the steps for training of Neural Network:

- Extract the features
- Pruning the feature dimension
- Split the feature sets into training and validation sets.
- Choose the network configuration which gives largest validation accuracy.
- In our case the model of the neural network has two hidden layers having 100 and 90 neurons respectively.
- Store the model.

## Classification and recognition

**Fig. 7:**
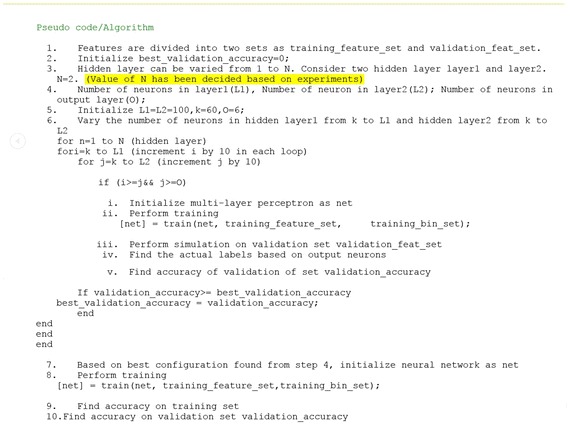
Pseudo code for selection of best architecture MLP

ANN in recent years has proved to be an advance tool in solving classification or pattern recognition problems.ANN is an efficient information processing system which resembles a biological neural network. ANNs are robust and fault tolerant in nature. They process information in parallel and they learn by examples. In this work, to recognize handwritten English character a multilayer perceptron has been used. The architecture used for Neural Network is arranged in layers and so the model is termed as multilayer perceptron. It consists of a layer of input nodes, one or more layers of hidden nodes, and one layer of output nodes. We have designed a function for the ANN classifier which provides us with optimization in terms of selecting the best structure for MLP. Figure 7 presents the pseudo code for selecting best structured MLP. The best structure includes number of hidden layers and number of neurons present in hidden layers. The best structure is found by calculating the accuracy on validation set in each iteration and storing the best one each time. The learning algorithm used in the network is back-propagation gradient descent algorithm.

Following are the steps for testing of Neural Network:

- Extract the features.
- Pruning the feature dimension
- Restore the model.
- Get the accuracy on test set.

### Experimental set up

During the phase of feature selection process a multi layered feed forward back-propagation neural network having two hidden layers with architecture 240-100-90-6 is used. The length of the feature vector decides the number of neurons in the input layer. The recognition system consists of a three-layered feed forward NN (Hanmandlu et al. 2003; Hanmandlu and Murthy 2007; Pradeep et al. 2011) with an input of 240 features extracted using:

- Box method
- Diagonal distance approach
- Mean
- Gradient operations approach
- Standard deviation
- Centre of gravity
- Edge detection

There are 52 characters (26 for small and 26 for capital alphabets) to be classified. Hence 6 bit variable is required for the output. Thus the number of output neurons is six. Log sigmoidal (logsig) is the activation function for neurons in hidden and output layers.

It is very difficult to choose the number of hidden layers and number of neurons in hidden layer in the architecture of neural network. Generally, for most of the applications, one hidden layer is enough, but the best way to choose the number of neurons and number of hidden layer is experimentation. Typically number of neurons in a hidden layer is determined by a combination of previous expertise, amount of data available, dimensionality, complexity of the problem, trial and error or validation on an additional training dataset (Polikar 2006). In our work different Neural Networks have been trained with different numbers of hidden layer and hidden neurons and measure the performance of those networks as explained in Fig. 7.

**Fig. 8:**
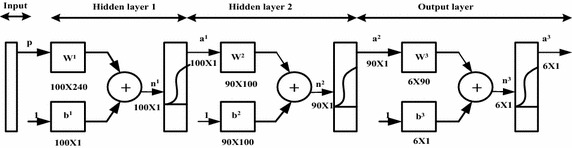
Three layer neural network

We retained the configuration that yield the best performing network. The accuracy on validation set saturated for the configuration chosen. Table 5 shows the best MLP architecture for the feature selection and Table 6 shows the training parameters for the feature selection that produces the best results in our case. Figure 8 shows the structure of feed-forward neural network.

**Table 5:** MLP architecture for feature selection

No. of input nodes	No. of hidden layers	No. of nodes in hidden layer 1	No. of nodes in hidden layer 2	No of output nodes
240	2	100	90	6

**Table 6:** The training parameters of MLP for feature selection

Training algorithm	Performance function	Training epochs	Learning rate	Momentum	Training goal
traingdx	Mean square error	1000 or till convergence	Adaptive	0.9	0.0001

The structure of the MLP is adaptive in nature. During the classification phase of the proposed work the number of input layer neurons have been changed to 76 as the number of features have been reduced to 76 after the implementation of GA and rest of the architecture of the MLP remains same.

## Result and discussion

The architecture of the neural network consists of an input layer, two hidden layers and an output layer. The number of neurons are 240, 100, 90 and 6 respectively for input, two hidden and output layer respectively during the feature selection and the number of input neurons during classification stage is 76. The neural network is trained using gradient descent back-propagation algorithm with momentum and adaptive learning rate. The log sigmiodal activation function is used to calculate the output. 19,145 known dataset of alphabet is used to train the network. After training the network, the system was tested using 2183 unknown alphabet dataset. In this proposed recognition system seven different approaches of feature extraction have been used. Feature selection is mostly done by heuristic or by intuition for specific type of character recognition application. The results are summarised in Tables 7, 8, 9, 10, 11 and 12. As seen from the Table 7, the number of features has been reduced to 76 by using GA. Table 8 shows the final features combination that provides the best results. It has been observed in practice that the feature selection process results in reduced features with slightly degradation in performance (Kim and Kim 2000). Hence the process of feature selection is applied where efficiency in terms of speed along with space requirement are important, despite the recognition accuracy been deteriorated. This specially happens in case of small features dimensional problem where as for larger dimensional feature space accuracy improves. Table 9 shows the accuracy of the proposed system is 94.65 % for capital alphabet and 91.11 % for small alphabet and it also shows that the accuracy of the proposed system is greater than the original one, where all the features have been considered.

**Table 7:** Number of features

Networks	Number of features
Adaptive MLP classifier without feature reduction	240
Adaptive MLP classifier with feature reduction	76

**Table 8:** Final feature combination for best results

Feature set	Number of features
Box features	23
Diagonal distance	7
Mean	9
Gradient operations	13
Standard deviation	12
Centre of gravity	8
Edge detection	4

**Table 9:** % Accuracy

Networks	% Accuracy for capital alphabet	% Accuracy for small alphabet
Adaptive MLP classifier without feature reduction	91.56	87.49
Adaptive MLP classifier with feature reduction	94.65	91.11

**Table 10:** Computational time

Networks	Testing time (m sec)
Adaptive MLP classifier without feature reduction	43
Adaptive MLP classifier with feature reduction	25

**Table 11:** Comparisons with other methods without feature selection

Authors	Dataset	Feature extraction method	Classifier	% Accuracy capital alphabet	% Accuracy small alphabet
Murthy and Hanmandlu (2011)	Samples From Matlab	Directional features	Fuzzy logic	83.85	
Nasien et al. (2010)	NIST	Freeman chain code	Support vector machine	88.46	86.00
Ali et al. (2010)	–	Wavelet compression	Euclidean distance	89.68	
Hanmandlu et al. (1999)	–	Fusion method	Neural network	86.00	
Singh and Hewitt (2000)	CEDAR	Hough transform	LDA Nearest neighbour	67.3 63.5	
Vamvakas et al. (2009)	CEDAR	Structural features	SVM	80.19	
Ganpathy and Liew (2008)	Self Created	–	Multiscale neural network	87.22	
Yuan et al. (2012)	UNIPEN		Convolution Neural network	93.7	90.2
Our approach	CEDAR	Hybrid features	Neural network	91.56	87.49

**Table 12:** Comparison with other methods using feature selection

Authors	Dataset	Feature extraction method	Feature selection	Classifier	% Accuracy capital alphabet	% Accuracy small alphabet
Chung and Yoon (1997)	NIST	Gradient	PCA	Neural network	93.31	–
		UDLRH			90.85	–
De Stefano et al. (2013)	NIST	Hybrid features	GA	SVM	57.88	
				MLP	62.57	
				KNN	61.45	
Proposed method	CEDAR	Hybrid features	GA	MLP NN	94.65	91.11

The proposed system performs well as it uses less number of features and implements an adaptive MLPNN classifier. Feature selection using GA is more proficient for features with larger dimension (240 in our case) than smaller dimension. This shows that redundant features are negatively contributing in accuracy of the classifiers.

As shown in Table 10, the computational time to recognize the test samples of handwritten character recognition has been reduced from 43 to 25 ms as the number of features reduces from 240 to 76. And also the proposed system requires less storage space. Reduction in features dimension is important as it improves the recognition speed (Kim and Kim 2000).

Comparisons of our handwritten character recognition method without using feature selection methodology with other existing methods which have not used feature selection have been shown in Table 11. Here an attempt is made to attain high accuracy using an adaptive MLPNN classifier. It is clear from the results that our method outperforms the other state of art methods with an accuracy of 91.56 and 87.49 % respectively for capital alphabet and small alphabet respectively except the work presented by Yuan et al. (2012). The details and number of training and testing set are not clear in their work but in our case we have considered whole training and testing dataset in the experiment. This superior adaptive structured MLP performance is due to the superior generalization ability of MLP in high dimensional space.

Table 12 gives the comparative analysis of the works which are using different feature selections techniques. It is evident from the results that the proposed method which implies Genetic Algorithm for feature selection gives better results compared to other existing methodologies.

## Conclusions

This paper presents hybrid feature extraction and GA based feature selection for off-line handwritten character recognition by using adaptive MLPNN classifier for achieving an improved overall performance on real world recognition problems. Seven approaches of feature extraction namely, box method, diagonal distance method, mean and gradient operation, standard deviation, centre of gravity and edge detection have been used to develop an off-line character recognition system. Two different recognition networks are built namely Adaptive MLP classifier without feature reduction and Adaptive MLP classifier with feature reduction. The network is trained and tested on the CEDAR CDROM-1 dataset. It can be concluded from the experimental results that the network which uses GA based feature selection method improves over all performance of the recognition system in terms of speed and storage requirement. It has also been verified that the proposed adaptive MLP Neural Network works as a better classifier and provides better accuracy.
